# Neural Network-Based Model Predictive Trajectory Tracking Control for Dual-Motor-Driven a Tracked Unmanned Vehicle

**DOI:** 10.3390/s25226877

**Published:** 2025-11-11

**Authors:** Li Zhai, Ye Yao, Jianghaoyu Yan, Chengping Wang, Chang Liu, Zhiquan Qi

**Affiliations:** 1 National Engineering Research Center for Electric Vehicle, Beijing Institute of Technology, Beijing 100081, China; zhaili26@bit.edu.cn (L.Z.); yaoye@bit.edu.cn (Y.Y.); 2 Shanghai Huawei Technologies Co., Ltd., Shanghai 201206, China; yanjianghaoyu@yinwang.com (J.Y.); wangchengping@yinwang.com (C.W.); 3 China Automotive Engineering Research Institute Intelligent Connected Technology Co., Ltd., Suzhou 215004, China; 3220210261@bit.edu.cn; 4 School of Mechanical Engineering, Beijing Institute of Technology, Beijing 100081, China

**Keywords:** tracked unmanned vehicle, trajectory tracking, model predictive control, Long Short-Term Memory (LSTM) network

## Abstract

Trajectory tracking is a key technology for electrical-driven tracked unmanned vehicles (TUVs), while the control model has a significant impact on tracking performance. To improve trajectory tracking accuracy for a dual-motor-driven TUV, a data-driven model-based predictive control scheme is proposed in this article. First, a vehicle dynamics model based on the Long Short-Term Memory (LSTM) network is developed for a TUV. The vehicle’s motion states in a subsequent time step are predicted using a sequence of history states and control inputs, while the multi-body dynamics model in the TUV platform are utilized for training and validation. Then, a neural network-based model predictive control (NN-MPC) strategy is designed, employing the trained LSTM model as the prediction model within a receding horizon framework to compute the optimal motor torques for trajectory tracking. Unlike existing learning-based MPC approaches that mainly focus on wheeled vehicles, this work investigates a neural network-enhanced MPC for tracked unmanned vehicles with coupled longitudinal–lateral dynamics. The simulation results demonstrate that, compared to a physics-model based MPC strategy, the proposed NN-MPC reduces the root mean square (RMS) values of lateral error and heading error by 12.1% and 7.9% in a medium-speed scenario and by 80% and 14.0% in a high-speed scenario. The field experiment further verifies the practical feasibility of the proposed control scheme.

## 1. Introduction

With the advantages of high load-carrying capacity, low ground-to-ground pressure, and zero-radius steering, tracked vehicles play an important role in modern military, agriculture, construction, mining, and disaster relief, as well as in response to emergencies [1]. Owing to their high efficiency, high power density, and flexible layout, electrical-driven tracked unmanned vehicles have become an important development direction for ground combat platforms [2]. Trajectory tracking, which aims to ensure vehicles track their predefined trajectories, has emerged as a key technology for unmanned vehicles [3]. Due to harsh driving conditions and complex road conditions, achieving high-precision trajectory tracking remains a significant challenge for tracked unmanned vehicles.

Many control approaches have been presented in trajectory tracking, such as the pure tracking algorithm [4], PID control [5], sliding mode control [6], linear quadratic regulator (LQR) [7], model predictive control (MPC) [8], and Deep Reinforcement Learning (DRL)-based methods [9]. Despite the concise structure of PID controllers, obtaining suitable control parameters and achieving robust performance remain challenging [10]. Although the optimal parameters can be derived by the LQR, it is difficult to deal with its constraints [11]. Due to the lack of interpretability, the stability of DRL continues to pose significant challenges [12]. Conversely, MPC exhibits strong capabilities in constraint handling, state prediction, and objective optimization, making it a promising approach for achieving accurate trajectory tracking control [13].

There have been many existing works on MPC-based trajectory tracking control. Chu et al. [14] employed an MPC controller based on a four-wheel kinematic model to achieve trajectory tracking control for passenger vehicles. Zhang et al. [15] presented a spatial-domain-based kinematic model and a linear MPC controller for path tracking. Although both MPC controllers achieve satisfactory tracking performance using the kinematic model, neglecting the side-slip effect leads to degraded control accuracy, especially in high-speed scenarios. To describe the vehicle dynamics characteristics more accurately, a single-track dynamic model was used in [16], which exhibited improved tracking performance during large steering maneuvers. In [17], a robust MPC scheme incorporating the single-track dynamic model was proposed to enhance robustness against parameter uncertainties and varying speeds. In [18], an event-trigger MPC framework is presented to reduce computational burden and improve real-time performance. Instead of focusing on wheeled vehicles, Chen et al. [19] proposed a dynamic-model-based MPC scheme for tracked vehicles to achieve trajectory tracking. In [20], an MPC-based strategy considering slipping and smoothing was proposed for an unmanned underwater tracked bulldozer to realize smooth motion control. Meanwhile, a tube–MPC method was developed in [21] for a substation inspection robot to enhance the robustness of trajectory tracking.

Despite these advancements, most MPC-based studies still rely on physics-based prediction models such as kinematic or dynamic models. However, these models often lead to performance degradation due to modeling mismatches between the prediction model and actual system behaviors [22]. In particular, for TUVs, the powertrain structure and steering mechanism differ significantly from wheeled vehicles. Steering is achieved by adjusting the velocity or torque between the two tracks [23], which results in coupled longitudinal–lateral dynamics and strong nonlinearities [24]. This makes accurate physics-based modeling challenging and may limit the achievable tracking performance. To address this problem, data-driven approaches, especially neural networks, have shown significant potential for representing complex nonlinear relationships [25]. For vehicle dynamic modeling tasks, system states are inherently time-dependent. As a special class of neural networks, the Long Short-Term Memory (LSTM) network is well suited for capturing long-term temporal dependencies and mitigating gradient vanishing issues compared to feed-forward neural networks and gated recurrent units (GRUs), making them an attractive alternative to physics-based models [26].

However, existing trajectory tracking control studies for TUVs still primarily adopt physics-based models [27,28]. Although acceptable trajectory tracking performance has been demonstrated, most approaches neglect coupled longitudinal–lateral dynamics, resulting in reduced precision and robustness under complex maneuvers. Moreover, the neural network-based dynamic modeling methods, as well as the integration of such models into an MPC framework for TUV trajectory tracking, have not been thoroughly investigated.

Motivated by the mentioned research gap, a data-driven-model-based predictive control scheme for a dual-motor-driven TUV is proposed in this article. The main contributions are as follows:

(1) A vehicle dynamics model based on a Long Short-Term Memory (LSTM) network is established for the dual-motor-driven TUV, incorporating coupled kinematic and dynamic characteristics. In this model, vehicle motion states in a subsequent time step are predicted using history states and control inputs.
(2) A novel MPC controller is developed for accurate trajectory tracking. Specially, the proposed LSTM-based vehicle dynamics model is employed to predict vehicle states in the receding horizon. The optimal motor torque is then calculated based on the prediction states and reference trajectory.

In addition, both cosimulations and field experiments are conducted to verify the effectiveness of the proposed scheme.

The remainder of this article is organized as follows: In Section 2, the LSTM-based vehicle dynamics model is constructed. In Section 3, the MPC-based trajectory tracking controller integrating the LSTM-based vehicle model is designed. In Section 4, the results of simulation and field experiment are demonstrated. The conclusions are drawn in Section 5.

## 2. Design of LSTM-Based Vehicle Dynamics Model

In this section, a data-driven vehicle dynamics model based on a LSTM network is introduced. Firstly, the model structure is demonstrated. Next, the process of data generation, model training, and validation are presented.

### 2.1. Model Structure and Input–Output Design

The vehicle states, such as position and velocity, exhibited pronounced time-series characteristics, demonstrating that the variation in vehicle states depends not only on the current control inputs, but also on the historical states and control commands. In this article, the vehicle states include the longitudinal and lateral positions, heading angle, the yaw rate, and longitudinal speed, all expressed in global coordinates. The control inputs are defined as *U* = [*T_L_*, *T_R_*]^T^, where *T_L_* and *T_R_* denote the motor torques applied to the left and right sides of the TUV, respectively.

**Figure 1:**
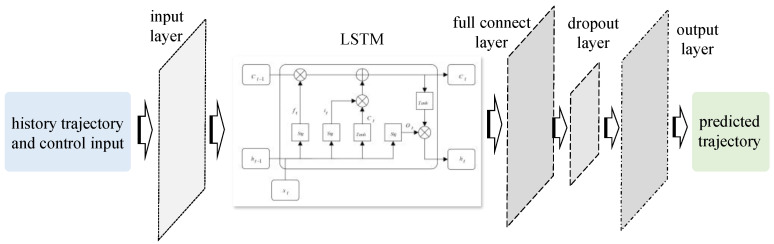
Network structure of the LSTM-based vehicle dynamics model for the TUV.

Due to the significant advantages of the LSTM network in processing time-series data [29], a LSTM-based vehicle dynamics model was established in which the vehicle states *X*_global_ at the subsequent time step were predicted using the fixed length of both the history states *X*_global_ and control inputs *U*. In this article, the LSTM network consists of an input layer, an LSTM layer, a fully connected layer (Dense layer), and a dropout layer. The overall model structure is shown in Figure 1.

Especially for the vehicle dynamics model shown in Figure 1, the fixed length of the historical states and control inputs of the input layer was set to 20, with a constant sampling interval 0.1 s. Assuming that *t* represents the current time slot, a data sequence consisting of vehicle states *X*_global_ and control inputs *U* at time slots *t −* 19, *t −* 18, …, *t* were fed into the LSTM layer from the input layer. The LSTM layer serves as the core component of the network, capturing long-term temporal dependencies between historical motion states and control inputs. A fully connected layer with ReLU activation maps the LSTM hidden representation to the output space. To reduce overfitting during training, a dropout layer was applied after the fully connected layer. Finally, the output layer produces the predicted vehicle states at the next time step, represented as follows:

(1) $$Y_{\mathrm{LSTM}}(t)=X_{\mathrm{global}}(t+1)={\left[X,Y,\varphi ,\omega ,v_{x}\right]}^{T}$$

where *X* and *Y* represent the longitudinal and lateral positions, *φ* is the heading angle, ω is the yaw rate, and *v_x_* is the longitudinal speed, all expressed in global coordinates.

For the network shown in Figure 1, the LSTM layer contains 32 hidden units. The number of parameters of the neural network vehicle dynamics model is summarized in Table 1.

### 2.2. Training and Validation of Neural Network Prediction Model

Based on the neural network model architecture, a multi-body dynamics model of a TUV in RecurDyn V9R4 was first used to obtain raw data for model training. The raw data was then pre-processed to match the network input dimensions. Subsequently, the constructed dataset was partitioned into training and testing subsets. The neural network model was trained using the training set, and its performance was evaluated on the test set. The prediction accuracy was quantified using the mean square error (MSE) between the predicted and actual values.

#### 2.2.1. Multi-Body Dynamics Modeling of TUV

The multi-body dynamics software RecurDyn enables highly accurate nonlinear dynamic simulations of complex tracked vehicle structures. It allows for direct application of rotational speed or torque to both sides of the wheels while providing vehicle state information, such as position, speed, track speed, acceleration, and yaw rate, for trajectory tracking control.

**Figure 2:**
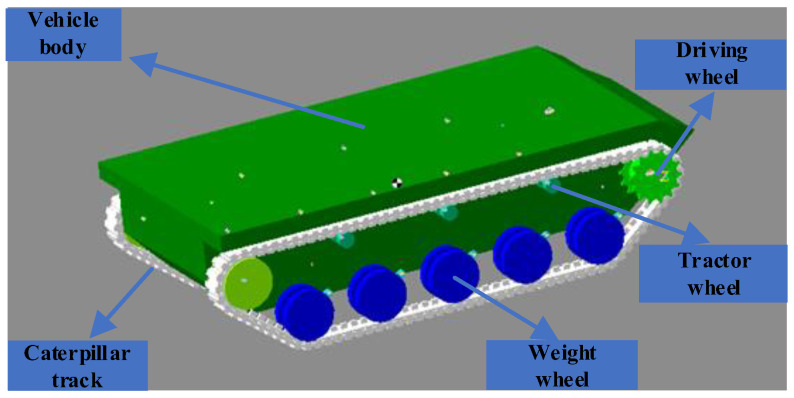
Multi-body dynamics model of TUV in RecurDyn.

The parameters of TUV in RecurDyn are shown in Table 2, while the simulated TUV in RecurDyn is shown in Figure 2.

#### 2.2.2. Data Acquisition and Preprocessing

For the established multi-body dynamics model in RecurDyn, the initial driving torque is manually set, and a random torque increment is subsequently applied to each side at each time step. The TUV state information in *X*_global_ is recorded at each step. A 1000 s simulation is performed, generating a raw dataset containing 50,000 data entries.

The main steps for preprocessing raw data are demonstrated as follows:

Step 1: Data cleansing

During data collection and recording, particularly in real vehicle experiments, anomalies may occur. Invalid data, outliers, and missing values must be removed or appropriately processed. Missing data are filled using the nearest-neighbor interpolation method, while outliers are identified and treated based on the 3*σ* principle. Data points exceeding the proximity threshold are removed. In addition, since vehicle state information is continuous, a filtering method is applied to suppress noise and smooth the data.

Step 2: Feature Normalization

Although the network structure applies scaling with the Sigmoid or tanh functions, the value ranges of different features are first normalized to a common scale. This ensures efficient training and preserves the contribution of features with smaller magnitudes, thereby preventing certain features from dominating the model learning process. The Min–Max normalization method is adopted, expressed as

(2) $$x_{\mathrm{norm}}=\frac{x-\min(x)}{\max(x)-\min(x)}$$

By processing the data through (2), the raw data is mapped to the interval [−1, 1] to achieve normalization.

Step 3: Serialization Processing

For the LSTM-based model, the time-series data is divided into multiple fixed-length segments, and each segment is assigned a corresponding target value at the final time step.

Step 4: Data division

The dataset is split into training, validation, and test sets for model training, optimization, and evaluation. It is noted that all training and validation data are collected from the RecurDyn cosimulation platform. The real-vehicle experimental data are not used for training, but only for validation of the control performance in field experiments, as detailed in Section 4.2. Initially, the original dataset is divided in a 7:3 ratio. Of the remaining 30%, 15% is used for validation and the rest for testing. To enhance model generalization and reduce overfitting, the training set is further divided into smaller batches, and a batch size of 64 is adopted for multiple training iterations.

#### 2.2.3. Training and Validation of the Vehicle Dynamic Model

Using the generated dataset, MSE is chosen as the loss function, which quantifies the difference between predicted and actual values, defined as

(3) $$\mathrm{MSE}=\frac{1}{n}\sum_{i=1}^{n}{\left(y_{i}-{\hat{y}}_{i}\right)}^{2}$$

where *y_i_* is the actual value, *ŷ_i_* is the predicted value and *n* is the sample size. The Adam optimizer is adopted with a learning rate of 0.005 and 200 training epochs.

Table 3 presents the prediction results of three models: a physics-based dynamic model in [24], a full-connected network DNN [30], and the proposed LSTM. The metric MAE represents mean absolute error. All neural models are trained and evaluated on the same dataset. For the DNN and LSTM, the results are averaged over ten independent runs for reliability. As depicted in Table 3, the LSTM model achieves the highest accuracy by effectively capturing the complex relationships between vehicle states. Although the DNN also has learning capability, its inability to handle long-term dependencies leads to reduced prediction accuracy. In contrast, the dynamic model exhibits the largest MSE and MAE, revealing its limitations in complex scenarios.

Furthermore, a parameter sensitivity analysis is conducted for the proposed LSTM model, where the candidate numbers of hidden units of LSTM layer are 16, 32, and 64 [26], and the candidate fixed length of historical states are 10, 20, and 30. Using MSE as the evaluation metric, the prediction results are listed in Table 4.

As shown in Table 4, increasing the number of hidden units and the length of historical states generally improves prediction accuracy, as more temporal dependencies are captured. However, excessively large hyperparameters result in slight performance degradation, likely due to overfitting and increased model complexity. Considering the trade-off between accuracy and model size, the optimal configuration of the LSTM network is determined to be 32 hidden units with a history length of 20 steps.

## 3. NN-MPC Trajectory Tracking Control Synthesis

**Figure 3:**
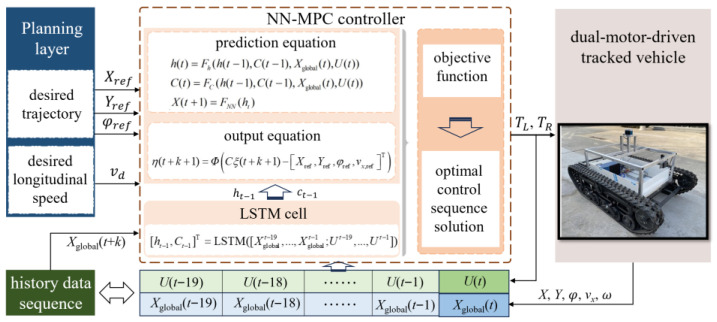
NN-MPC strategy for trajectory tracking.

In this section, a trajectory tracking controller based on the neural network model predictive control (NN-MPC) is designed to obtain the optimal motor torque. The prediction framework using the LSTM-based vehicle dynamics model is first demonstrated. Next, the objective function and solution decision is introduced. The stability analysis of NN-MPC is also discussed. The structural block diagram of the proposed control strategy is shown in Figure 3.

### 3.1. Establishment of NN-MPC Prediction Model

Directly embedding the neural network prediction model within the MPC framework causes the iterative updates of the state variables over the prediction horizon. Consequently, the state matrix also updates. This process involves overwriting the original data with new predictions, which complicates the optimization and may degrade the solution quality. Therefore, a carefully designed prediction model is required to ensure stability and efficient computation.

For the prediction equation, the dimensional transformations of the neural network can be formulated as

(4) $${Z^{\mathrm{in}}}_{20\times 7}\underset{\text{LSTM-layer}}{\Rightarrow }{Z^{1}}_{32\times 1}\underset{\text{Dense-layer}}{\Rightarrow }{Z^{2}}_{16\times 1}\underset{\text{Output-layer}}{\Rightarrow }{Z^{\mathrm{out}}}_{5\times 1}$$

The final network output is represented as Z^out^_5*1_ = *X*_global_(*t* + 1). Only the inputs to the LSTM layer are multidimensional matrices, while all of the inputs and outputs of subsequent layers are one-dimensional vectors. Within the LSTM layer, the sequential data passes through 20 LSTM units sharing the same network parameters, where 20 corresponds to the length of historical states. Each LSTM unit takes the predicted output from the previous time step and the corresponding original cell state sequence as the input, producing a new predicted output along with an updated cell state. This process corresponds to the update of LSTM cell state, which can be mathematically expressed as

(5) $${\left[h_{t},C_{t}\right]}^{T}=f_{\mathrm{LSTM}}(h_{t-1},C_{t-1},{\left[X_{\mathrm{global}}(t),U(t)\right]}^{T})$$

where *h_t_* and *C_t_* denote the hidden state and cell state of the LSTM layer at time step *t* [26], *h_t−_*_1_ and *C_t−_*_1_ represent their corresponding values at the previous time step *t −* 1.

It means that the predicted output *h_t_* of the LSTM at time *t* can be directly obtained by *h_t−_*_1_, *C_t−_*_1_, *X*_global_(*t*), *U*(*t*), while simultaneously producing *h_t_* and *C_t_* for the next iteration. Based on this concept, the LSTM layer with 20 history states in (5) is restructured into an LSTM layer, with 19 history states, and a Dense layer, with network parameters kept identical. Equation (5) can be written as

(6) $${Z^{\mathrm{in}}}_{20*7}\underset{\text{LSTM-layer}}{\Rightarrow }[h_{t-1},C_{t-1},X_{\mathrm{global}}^{t},U^{t}]\underset{\text{Dense-layer}}{\Rightarrow }{Z^{1}}_{32*1}\underset{\text{Dense-layer}}{\Rightarrow }{Z^{2}}_{16*1}\underset{\text{Output-layer}}{\Rightarrow }{Z^{\mathrm{out}}}_{5*1}$$

In this way, the original neural network prediction model can be regarded as a combination of a single-layer LSTM network that processes sequential data and a fully connected network that handles one-dimensional input. The resulting simplified single-layer neural network operates independently of the MPC and does not participate in state iterations within the prediction horizon.

The prediction of state variables can be expressed as follows:

(7) $$\left\{\begin{array}{l} {\left[h_{t-1},C_{t-1}\right]}^{T}=\mathrm{LSTM}([X_{\mathrm{global}}^{t-19},\ldots ,X_{\mathrm{global}}^{t-1};U^{t-19},\ldots ,U^{t-1}])\\ h(t)=F_{h}(h(t-1),C(t-1),X_{\mathrm{global}}(t),U(t))\\ C(t)=F_{C}(h(t-1),C(t-1),X_{\mathrm{global}}(t),U(t))\\ X(t+1)=F_{NN}(h_{t}) \end{array}\right.$$

where *F_h_* and *F_C_* represent the process of updating cell states and hidden states within a LSTM cell. *F_NN_* represents the function for computing the cell output. The detailed formulation of *F_h_*, *F_C_*, and *F_NN_* can be found in [26].

Based on (7), the prediction equation of hidden state *h*, cell state *C*, and vehicle motion state *X*_global_ at time slot *t* + *k* is given by

(8) $$h(t+k)=F_{h}(h(t+k-1),C(t+k-1),X_{\mathrm{global}}(t+k),U(t+k))$$

(9) $$C(t+k)=F_{C}(h(t+k-1),C(t+k-1),X_{\mathrm{global}}(t+k),U(t+k))$$

(10) $$X_{\mathrm{global}}(t+k+1)=F_{NN}(h(t+k))$$

Selecting *ξ*(*t*) = [*h_t−_*_1_, *C_t−_*_1_, $X_{\mathrm{global}}^{T}$]^T^ as the state variables and *U*(*t*) = [*T_R_*(*t*), *T_L_*(*t*)]^T^ as the control input, Equations (8)–(10) can be regarded as the prediction equation of NN-MPC. To improve computation efficiency, an approximate linearization is further performed around the hidden and cell states of the LSTM layer (*h_t−_*_1_, *C_t−_*_1_), current trajectory point X_global_, and previous control input *U*(*t −* 1). At time slot *t*, these quantities are all known from the previous iteration and sensor measurements. It is noted that the linearization is not performed at a fixed equilibrium point, but rather at the current trajectory point and corresponding LSTM states. Consequently, the resulting model is a local linear time-varying approximation corresponding to a trajectory-dependent linearization commonly adopted in NMPC design [31]. The linearized prediction model can be reconstructed as follows:

(11) $$\left\{\begin{array}{l} h(t+k)=A_{h}\xi (t+k)+B_{h}\sum_{j=0}^{\min(k,N_{C})}\Delta U(t+j)+H_{h}\\ C(t+k)=A_{C}\xi (t+k)+B_{C}\sum_{j=0}^{\min(k,N_{C})}\Delta U(t+j)+H_{C}\\ X_{\mathrm{global}}(t+k+1)=A_{X}\xi (t+k)+B_{X}\sum_{j=0}^{\min(k,N_{C})}\Delta U(t+j)+H_{X} \end{array}\right.$$

where *ξ*(*t* + *k*) = [*h_t+__k−_*_1_, *C_t+__k−_*_1_, $X_{\mathrm{global}}^{T}$(*t* + *k*)${]}^{T}$, and *N_C_* denotes the length of control horizon in the MPC design. The control input *U* is transformed into its control increment form ∆*U*, in which ∆*U*(*t*)= *U*(*t*) − *U*(*t* − 1). The matrices in (11) are given in (12) and (13).

(12) $$\begin{array}{lll} A_{h}=\left[{\left.\frac{\partial F_{h}}{\partial h}\frac{\partial h}{\partial t}\right|}_{t-1},{\left.\frac{\partial F_{h}}{\partial C}\frac{\partial C}{\partial t}\right|}_{t-1},\frac{\partial F_{h}}{\partial X}\frac{\partial X}{\partial t}\right], & B_{h}={\left.\frac{\partial F_{h}}{\partial U}\frac{\partial U}{\partial t}\right|}_{t-1}, & B_{C}={\left.\frac{\partial F_{C}}{\partial U}\frac{\partial U}{\partial t}\right|}_{t-1}\\ A_{C}=\left[{\left.\frac{\partial F_{C}}{\partial h}\frac{\partial h}{\partial t}\right|}_{t-1},{\left.\frac{\partial F_{C}}{\partial C}\frac{\partial C}{\partial t}\right|}_{t-1},\frac{\partial F_{C}}{\partial X}\frac{\partial X}{\partial t}\right], & A_{X}=\frac{\partial F_{NN}}{\partial h}\frac{\partial h}{\partial t}A_{h}+I, & B_{X}=\frac{\partial F_{NN}}{\partial h}\frac{\partial h}{\partial t}B_{h} \end{array}$$

(13) $$\begin{array}{l} H_{h}=F_{h}\left(h(t-1),C(t-1),X_{\mathrm{global}}(t),U(t-1)\right)-A_{h}{\left(h_{t-1},C_{t-1},X_{\mathrm{global}}{\left(t\right)}^{T}\right)}^{T}-B_{h}U(t-1)\\ H_{C}=F_{C}\left(h(t-1),C(t-1),X_{\mathrm{global}}(t),U(t-1)\right)-A_{C}{\left(h_{t-1},C_{t-1},X_{\mathrm{global}}{\left(t\right)}^{T}\right)}^{T}-B_{C}U(t-1)\\ H_{X}=F_{X}\left(h(t-1),C(t-1),X_{\mathrm{global}}(t),U(t-1)\right)-A_{X}{\left(h_{t-1},C_{t-1},X_{\mathrm{global}}{\left(t\right)}^{T}\right)}^{T}-B_{X}U(t-1) \end{array}$$

Equation (11) can also be given by

(14) $$\xi (t+k+1)=A\xi (t+k)+B\sum_{j=0}^{\min(k,N_{C})}\Delta U(t+j)+H$$

where *A*, *B*, and *H* are given as

(15) $$A={\left[{A_{h}}^{T}\;{A_{C}}^{T}\;{A_{X}}^{T}\right]}^{T},B={\left[{B_{h}}^{T}\;{B_{C}}^{T}\;{B_{X}}^{T}\right]}^{T},H={\left[{H_{h}}^{T}\;{H_{C}}^{T}\;{H_{X}}^{T}\right]}^{T}$$

Although the future state *ξ*(*t* + *k*) and control input ∆*U*(*t + j*) appear in (14), they are not known a priori. Instead, the future states are recursively predicted from the current state *ξ*(*t*) and the sequence of control increments ∆***U***. Specifically, ∆***U*** is the decision variable consisting of ∆*U*(*t*), ∆*U*(*t* + 1), …, ∆*U*(*t* + *N_C_ −* 1) in the NN-MPC. Consequently, (14) can be equivalently transformed into a formulation that depends only on the current state *ξ*(*t*) and the decision variable ∆***U***, which ensures strict causality in the optimization.

As mentioned above, the linearization process is carried out around current trajectory point X_global_ and previous control input *U*(*t −* 1), leading to a local linear time-varying approximation. The matrices *A* and *B* in (14) are obtained from the Jacobians of the NN-based prediction model with respect to the linearization point. Since the linearization is performed online at each current trajectory point, and the actual vehicle states remain within a small neighborhood of this point during operation, it is reasonable to assume that the pair (A,B) obtained from this local linearization is locally controllable within a compact neighborhood of the linearization point. Furthermore, the control sequence is computed by solving an optimization problem in the MPC design. Since the cost function is quadratic and the constraints are convex, the MPC optimization problem has a unique feasible solution within the local admissible region, thereby ensuring the feasibility of the resulting control action.

Choosing *η* = [*y_e_*, *φ_e_*, *v_e_*]^T^ as the output variable, where *y_e,_ φ_e_*, and *v_e_* denote lateral error, heading error and speed tracking error. The value of *η* at time slot *t* + *k* + 1 is given by

(16) $$\begin{array}{l} \eta (t+k)=\Phi \left(C\xi (t+k)-S_{\mathrm{ref}}(t+k)\right)\\ s.t.\;S_{\mathrm{ref}}(t+k)={\left[X_{\mathrm{ref}}(t+k),Y_{\mathrm{ref}}(t+k),\varphi_{\mathrm{ref}}(t+k),v_{x,\mathrm{ref}}(t+k)\right]}^{T} \end{array}$$

where Φ and *C* are represented as

(17) $$\Phi =\left[\begin{array}{cccc} \sin\left(\varphi_{\mathrm{ref}}(t)\right) & \cos\left(\varphi_{\mathrm{ref}}(t)\right) & 0 & 0\\ 0 & 0 & 1 & 0\\ 0 & 0 & 0 & 1 \end{array}\right],\;C=\left[\begin{array}{ccccccc} 0 & 0 & 1 & 0 & 0 & 0 & 0\\ 0 & 0 & 0 & 1 & 0 & 0 & 0\\ 0 & 0 & 0 & 0 & 1 & 0 & 0\\ 0 & 0 & 0 & 0 & 0 & 0 & 1 \end{array}\right]$$

By combining formulas (14) and (16), the prediction model of NN-MPC is obtained as follows:

(18) $$\left\{\begin{array}{l} \xi (t+k+1)=A\xi (t+k)+B\sum_{j=0}^{\min(k,N_{C})}\Delta U(t+j)+H\\ \eta (t+k)=\Phi \left(C\xi (t+k)-S_{\mathrm{ref}}(t+k)\right) \end{array}\right.$$

### 3.2. Objective Function and Optimal Solution

The output equation of the neural network prediction model at moment *t* is expressed as follows:

(19) $$Y(t)=\Psi \left(C_{F}\left(A_{F}x(t)+B_{F}\Delta U(t)+D_{F}H(t)\right)-S_{F}\right)$$

where the matrices ***A_F_***, ***B_F_***, ***C_F_***, ***D_F_***, ***S****_F_*, and Ψ are given in (20)–(22).

(20) $$A_{F}=\left[\begin{array}{c} A\\ \vdots \\ A^{N_{C}}\\ \vdots \\ A^{N_{P}} \end{array}\right],\;B_{F}=\left[\begin{array}{ccc} B & \ldots & 0\\ \vdots & \ddots & \vdots \\ \sum_{i=0}^{N_{C}-1}A^{i}B & \ldots & B\\ \vdots & & \vdots \\ \sum_{i=0}^{N_{P}-1}A^{i}B & \ldots & \sum_{i=0}^{N_{P}-N_{C}}A^{i}B \end{array}\right],\;C_{F}=\underset{N_{p}\;\mathrm{diagonal}\;\mathrm{elements}\;}{\underset{⏟}{\left[\begin{array}{cccc} C & & & \\ & C & & \\ & & \ddots & \\ & & & C \end{array}\right]}},D_{F}=\left[\begin{array}{c} I\\ \vdots \\ A^{N_{C}-1}\\ \vdots \\ A^{N_{P}-1} \end{array}\right]$$

(21) $$S_{F}=\left[\begin{array}{c} S_{\mathrm{ref}}(t+1)\\ \vdots \\ S_{\mathrm{ref}}(t+N_{C})\\ \vdots \\ S_{\mathrm{ref}}(t+N_{P}) \end{array}\right],\;\Psi =\underset{N_{p}\;\mathrm{diagonal}\;\mathrm{elements}\;}{\underset{⏟}{\left[\begin{array}{cccc} \Phi & & & \\ & \Phi & & \\ & & \ddots & \\ & & & \Phi \end{array}\right]}}$$

(22) $$Y(t)={\left[\eta {\left(t+1|t\right)}^{T},\ldots ,\eta {\left(t+N_{P}|t\right)}^{T}\right]}^{T},\;\;\Delta U(t)={\left[\Delta U{\left(t|t\right)}^{T},\ldots ,\Delta U{\left(t+N_{C}-1|t\right)}^{T}\right]}^{T}$$

where *N_P_* is the length of the prediction horizon.

The objective function of trajectory tracking control based on the neural network prediction model is expressed as follows:

(23) $$\underset{\Delta U}{\min J}=\sum_{i=1}^{N_{p}}{\left\|\eta (t+i|t)\right\|}_{Q}^{2}+\sum_{i=1}^{N_{c}-1}{\left\|\Delta U(t+i|t)\right\|}_{R}^{2}+\sigma \varepsilon^{2}$$

where the weight matrix for tracking error ***Q*** = diag([*Q_ye_*, *Q_φe_*, *Q_ve_*]), *Q_ye_*, *Q_φe_*, *Q_ve_* are the weight matrix for lateral error, yaw error, and speed error. *R* is the weight matrix for the control effort, which is characterized by the deviation of the control input. ε is the slack variable and σ is a positive weight for ε.

The constraints are established, and the constraints on the state variables are expressed as follows:

(24) $$\omega_{\min}\leq \omega \leq \omega_{\max},\;\mathrm{where}\;\left|\omega_{\min}\right|=\left|\omega_{\max}\right|=\left|v_{x}\rho \right|$$

The constraints on the output variables are expressed as follows:

(25) $$y_{e\_\min}\leq y_{e}\leq y_{e\_\max},\;\mathrm{where}\;\left|y_{e\_\min}\right|=\left|y_{e\_\max}\right|=1.5m$$

(26) $$\varphi_{e\_\min}\leq \varphi_{e}\leq \varphi_{e\_\max},\;\mathrm{where}\;\left|\varphi_{e\_\min}\right|=\left|\varphi_{e\_\max}\right|=0.52$$

The constraints on the control variables are given by

(27) $$\left\{\begin{array}{l} T_{R\min}\leq T_{R}\leq T_{R\max}\\ T_{L\min}\leq T_{L}\leq T_{L\max} \end{array}\right.,\mathrm{where}\;{\left|T_{i}\right|}_{\max}=\min(T_{m},9549\frac{P_{m}}{n_{i}})$$

where *P_m_* and *n_i_* are motor power and speed.

To enhance the stability of the closed-loop system, the constraint for terminal state is given as [32]

(28) $$\eta (t+N_{p}|t)=0$$

Based on all the above constraints, Equation (30) is simplified to the standard form of quadratic programming, represented as follows:

(29) $$\left\{\begin{array}{l} \underset{\Delta U\in R^{m}}{\min}J(\Delta U)=\frac{1}{2}\Delta U^{T}\Theta \Delta U+\Psi \Delta U\\ s.t.\;\;\;y_{e\_\min}\leq y_{e}\leq y_{e\_\max},\varphi_{e\_\min}\leq \varphi_{e}\leq \varphi_{e\_\max},\;\omega_{\min}\leq \omega \leq \omega_{\max}\\ \;\;\;\;\;\;\;\;\left|T_{R}\right|\leq {\left|T_{R}\right|}_{\max},\left|T_{L}\right|\leq {\left|T_{L}\right|}_{\max},\;\eta (t+N_{p}|t)=0 \end{array}\right.$$

The optimal ∆***U*** was obtained, and its first element was added to the previous desired motor torque, yielding *U*(*t*) = *U*(*t −* 1) + ∆*U*(*t*). *U*(*t*) serves as the torque command transmitted to the actuator of the TUV.

Since the TUV is an underactuated system, it possesses nontrivial zero dynamics. In the proposed NN-MPC framework, the control inputs are generated by solving an optimization problem that includes the lateral, heading, and velocity tracking errors with corresponding weighting coefficients. These weights enable the controller to dynamically balance multiple tracking objectives and generate feasible control torques that drive the vehicle along the desired trajectory. It should be emphasized that, in real-world trajectory tracking applications, the primary objective is also to achieve satisfactory path-following performance (e.g., acceptable position, heading, and velocity error, rather than strict asymptotic convergence of all states).

In addition, due to the non-holonomic constraint of the TUV, the lateral axis is not directly controllable when the TUV is stationary. In such special conditions, this issue can be handled through the coordination of the overall control architecture. Specifically, when the TUV is stationary, the upper-level trajectory planning module replans the desired trajectory by taking the current vehicle position as the starting point. Once the vehicle starts moving, the NN-MPC controller dynamically adjusts the control torques to minimize the tracking errors. When the vehicle comes to a stop, the tracking process is considered complete if the terminal errors remain within acceptable tolerance bounds rather than absolute zero error, which is a standard practice in trajectory tracking control.

### 3.3. Stability Analysis

The stability of the proposed MPC controller is demonstrated in this subsection. The stability analysis for the nominal NN-based model is first presented, and then the practical stability under the modeling error caused by the NN-based model is discussed. It is noted that the focus of this article is primarily on practical implementation and control effectiveness, while the theoretical stability analysis is included here to ensure completeness.

#### 3.3.1. Stability Analysis with Nominal NN-Based Model

For the stability of the nominal model, it is assumed that the NN-based model is sufficiently accurate. A candidate Lyapunov function $J^{*}$ is formulated as

(30) $$J^{*}(t)=\min J(t),\;\mathrm{where}\;J(t)=\sum_{i=1}^{N_{p}}\left({\left\|\eta (t+i|t)\right\|}_{Q}^{2}+{\left\|\Delta U(t+i-1|t)\right\|}_{R}^{2}\right)$$

where *t* is time slot, η and ΔU are consistent with those presented in (22). It is evident that $J^{*}\geq \;$0, while $J^{*}=\;$0 only if η(*t* + *i*|*t*) = 0 and ΔU(*t* + *i −* 1|*t*) = 0 for all *i*, which means that $J^{*}$ is positive-definite.

For the terminal constraint presented in (28), the initial feasibility is assumed to hold, i.e., there exists a feasible control sequence increment ∆***U***(0) such that η(*N_P_*|0) = 0. The assumption is reasonable, since the initial feasibility can be ensured through coordination among different modules of the tracked unmanned vehicle (TUV). For example, the upper-layer trajectory planning module generates the desired trajectory consistent with the initial position and speed of the TUV, ensuring feasible initialization of the control process.

Then, the recursive feasibility is discussed. Without loss of generality, the feasibility of terminal constraint at time slot *t* + 1 is analyzed, assuming that the feasibility of terminal constraint $\eta (t+N_{p}|t)=0$ is satisfied at time slot *t*.

Due to the feasibility of $\eta (t+N_{p}|t)=0$, it is assumed that, at time slot *t*, a control sequence increment ∆***U_p_***(*t*) exists that drives the predicted state to zero within *N_P_* steps. The ∆***U_p_***(*t*) is given as

(31) $$\Delta U_{p}(t)={\left[\Delta U_{p}(t|t),\Delta U_{p}(t+1|t),\ldots ,\Delta U_{p}(t+N_{p}-1|t)\right]}^{T}$$

Applying the ∆*U**_p_***(*t*|*t*) as the current control input increment, the next-step state η(*t* + 1|*t*) is determined by the system model in (19) and ∆*U**_p_***(*t*|*t*). Therefore, there exists a feasible control sequence ∆***U_p_***(*t* + 1) satisfying $\eta (t+1+N_{p}|t)=0$. ∆***U_p_***(*t +* 1) is given by

(32) $$\Delta U_{p}(t+1)={\left[\Delta U_{p}(t+1|t),\ldots ,\Delta U_{p}(t+N_{p}-1|t),0\right]}^{T}$$

Therefore, the recursive feasibility is guaranteed if initial feasibility is satisfied, which implies that the terminal constraint remains valid for all subsequent time steps under the optimal control law.

For (30), define the optimal solution at time slot *t* as

(33) $$\Delta U_{t}^{*}={\left[\Delta U^{*}(t|t),\Delta U^{*}(t+1|t),\ldots ,\Delta U^{*}(t+N_{p}-1|t)\right]}^{T}$$

Then, $J^{*}(t)$ is given as

(34) $$J^{*}(t)=\sum_{i=1}^{N_{p}}\left({\left\|\eta^{*}(t+i|t)\right\|}_{Q}^{2}+{\left\|\Delta U^{*}(t+i-1|t)\right\|}_{R}^{2}\right)$$

where $\eta^{*}(t\;+\;i|t)$ is the output state under $\Delta U^{*}$.

Since the optimal solution in (33) is also a feasible solution satisfying the terminal constraint, there exists a feasible solution ∆***U*** (*t* + 1) at time slot *t* + 1, given as

(35) $$\Delta U(t+1)={\left[\Delta U^{*}(t+1|t),\ldots ,\Delta U^{*}(t+N_{p}-1|t),0\right]}^{T}$$

Based on (32) and (35), $J^{*}(t\;+\;1)$ satisfies

(36) $$\begin{array}{ll} J^{*}(t+1) & \leq J(t+1)\\ & =\sum_{i=1}^{N_{p}}\left({\left\|\eta (t+1+i|t+1)\right\|}_{Q}^{2}+\|\Delta U(t+i|t+1){\|}_{R}^{2}\right)\\ & =\sum_{i=1}^{N_{p}-1}\left(\|\eta^{*}(t+i+1|t){\|}_{Q}^{2}+\Delta U^{*}(t+i|t){\|}_{R}^{2}\right) \end{array}$$

where J(t + 1) is a feasible function value with ΔU(*t* + 1).

According to (34) and (36), we obtain

(37) $$J^{*}(t+1)-J^{*}(t)\leq -\|\eta^{*}(t+1|t){\|}_{Q}^{2}-\|\Delta U^{*}(t|t){\|}_{R}^{2}$$

Therefore, $J^{*}$ is a monotonically non-increasing function, and the stability of the proposed MPC controller is proved. It is noted that the terminal constraint in (28) and the assumption of initial feasibility are theoretical design tools commonly adopted in the MPC literature to establish closed-loop stability [31]. In practical implementations, this hard terminal constraint can be relaxed by introducing a terminal cost with sufficiently large weight, which effectively converts it into a soft constraint. This approach avoids infeasibility while maintaining stability in practice. Similarly, the initial feasibility can be ensured through coordination among the different modules of the tracked unmanned vehicle (TUV). For example, when the TUV is stationary, this condition can be regarded as an initial state. Although the lateral axis (*y*-axis) is not directly controllable for a differential-driven tracked vehicle when the vehicle is stationary, the upper-layer trajectory planning module can then replan a feasible trajectory by taking the current position of the TUV as the starting point, ensuring that the reference trajectory always satisfies the non-holonomic constraints. Consequently, the NN-MPC controller is only applied in regions where the system remains locally controllable.

#### 3.3.2. Practical Stability Under Bounded Model Error

The above proof assumes initial feasibility and neglects model mismatch between the NN-based model and the real system. In practice, the NN-based prediction model inevitably introduces the approximation error. The true system dynamics can be expressed as

(38) $$\xi_{tr}(t+1|t)=f_{NN}\left(\xi (t),\Delta U(t)\right)+e_{t},\;s.t.\;\left\|e_{t}\right\|\leq \rho$$

where *f_NN_* represents the nominal NN-based model and *ξ_tr_*(*t* + 1|*t*) is the true value of state *ξ*(*t* + 1|*t*). *e_t_* is the model error with the assumed upper bound ρ.

Since the output state η(*t* + 1|*t*) is derived from the predicted state *ξ*(*t* + 1|*t*), the modeling error between the true state *ξ_tr_*(*t* + 1|*t*) and predicted state *ξ*(*t* + 1|*t*) leads to a deviation between the corresponding output η(*t* + 1|*t*) and η*_tr_*(*t* + 1|*t*), expressed as

(39) $$\eta_{tr}(t+1|t)\leq \eta (t+1|t)+\delta ,\;s.t.\;\left|\delta \right|\leq C_{1}\rho$$

where the upper bound of deviation depends on the ρ. *C*_1_ is a positive constant determined by the mapping from system state *ξ*(*t* + 1|*t*) to the output η(*t* + 1|*t*).

Defining *J_tr_*(*t* + 1) and *J* (*t* + 1) as the true cost function value and nominal function value corresponding to η*_tr_*(*t* + 1|*t*) and η(*t* + 1|*t*), under the assumption that the cost function defined in (30) is locally Lipschitz, the deviation between *J_tr_*(*t* + 1) and *J* (*t* + 1) is given by

(40) $$\left\|J_{tr}(t+1)-J(t+1)\right\|\leq C_{2}C_{1}\rho$$

where *C*_2_ is a positive constant associated with local Lipschitz property of the cost function.

According to the stability analysis of nominal NN-based model and (38)–(40), we obtain

(41) $$\begin{array}{ll} \left\|J_{tr}(t+1)-J(t)\right\| & \leq \left\|J_{tr}(t+1)-J(t+1)\right\|+\left\|J(t+1)-J(t)\right\|\\ & \leq -\|\eta (t+1|t){\|}_{Q}^{2}-\|\Delta U(t|t){\|}_{R}^{2}+C_{2}C_{1}\rho \end{array}$$

Due to the boundness of control input and output η(*t* + 1|*t*), there exist positive constants $\alpha_{1}$ and $\alpha_{2}$ satisfying that

(42) $$\alpha_{1}\|\eta (t|t){\|}_{Q}^{2}\;\leq \;\|\eta (t+1|t){\|}_{Q}^{2}+\|\Delta U(t|t){\|}_{R}^{2}\;\leq \;\alpha_{2}\|\eta (t|t){\|}_{Q}^{2}$$

Substituting (42) into (41), we obtain

(43) $$\left\|J_{tr}(t+1)-J(t)\right\|\leq -\lambda_{\min}(Q)\times \alpha_{1}\|\eta (t|t){\|}^{2}++C_{2}C_{1}\rho$$

where λ_min_(*Q*) is minimum eigen value of the positive-definite matrix *Q*. Based on (43), it is evident that when $\left\|\eta (t|t)\right\|$ exceeds the boundary $\sqrt{C_{2}C_{1}\rho }/(\lambda$_min_(*Q*)$\times \alpha_{1}$), the actual cost function decreases monotonically, implying that the closed-loop system is ultimately bounded within a neighborhood of radius *O*($\sqrt{\rho }$) under the local Lipschitz assumption.

## 4. Simulation and Experiment Results

In this section, a cosimulation is first conducted in which the algorithms are implemented in Matlab/Simulink 2021b with the high-fidelity TUV model constructed using RecurDyn. Then, a field experiment is carried out on a scaled dual-motor-driven TUV.

### 4.1. Simulation Results and Discussion

**Figure 4:**
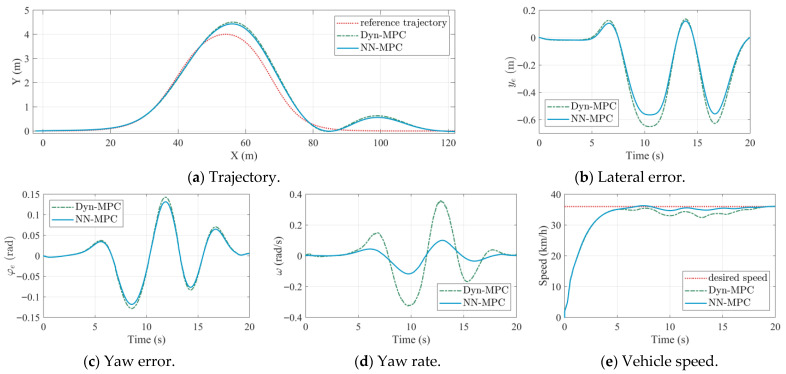
Comparison results of Dynamic-MPC and NN-MPC under medium speeds.

**Figure 5:**
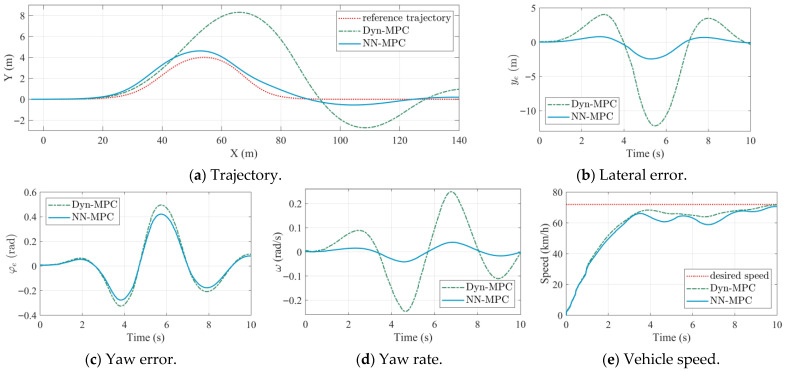
Comparison results under high speeds.

The proposed NN-MPC strategy is first evaluated in a medium-speed (36 km/h) and high-speed (72 km/h) double-lane-change scenarios. The MPC strategy using the dynamic model (Dyn-MPC) [28] is employed as the baseline. The controller parameters are listed in Table 5, where these parameters were tuned empirically to balance tracking accuracy and control smoothness. NN-MPC and Dyn-MPC use identical parameter settings to ensure a fair comparison. The simulation results under medium-speed and high-speed settings are shown in Figure 4 and Figure 5.

In Figure 4, the abscissa unit of the trajectory plot is meters, whereas the abscissa unit of other subplots is seconds. In medium-speed scenarios, the physical-based dynamic model can accurately capture the dynamic characteristics of the TUV. Subsequently, the actual path of the TUV under Dyn-MPC is almost consistent with that of NN-MPC, as shown in Figure 4a. Similarly, the lateral error and the yaw error under two approaches are very close, as shown in Figure 4b,c. However, due to the consideration of coupled longitudinal and lateral dynamics, NN-MPC outperforms in speed tracking compared to Dyn-MPC, as shown in Figure 4e. As depicted in Figure 4d, the response of yaw rate is also smoother for NN-MPC, which indicates a higher handling stability margin. This demonstrates the effectiveness of the LSTM-based vehicle dynamic model.

The performance metrics of trajectory tracking under the medium-speed scenario are listed in Table 6, in which RMS represents the root mean square value and MA represents the maximum value. The subscripts *l* and *h* denote the lateral error and heading error. As shown in Table 6, NN-MPC outperforms Dyn-MPC in both lateral and heading tracking accuracy. Compared with Dyn-MPC, the RMS values of lateral error and heading error decrease by 12.1% and 7.9%, respectively, while the MA values decrease by 13.1% and 7.5%.

For the high-speed scenario, due to the zero initial speed and the short straight segment on the desired path, combined with the limitations of motor power and tire–ground adhesion, the TUV is unable to reach the target high speed of 72 km/h before entering the curve segment, as shown in Figure 5a,e. Once the turning maneuver began, the coupled longitudinal–lateral dynamics led to noticeable speed oscillations under steering, resulting in a slight speed deficit relative to the desired value. After exiting the curve and returning to the straight segment, the vehicle continued to accelerate and gradually approached the target speed. Although a slight speed deviation existed during steering maneuvers, the proposed LSTM-based vehicle dynamic model and data-driven MPC strategy still achieved significantly improved lateral tracking accuracy, as shown in Figure 5b, demonstrating their effectiveness even at high speeds. Therefore, the limitation in achieving the target speed does not compromise the validity of the control performance evaluation. Nevertheless, more accurate motion control considering the coupling among longitudinal speed tracking, lateral path following, and handling stability coordination will be investigated in future work.

The performance metrics of trajectory tracking under the high-speed scenario are listed in Table 7. As shown in Table 7, NN-MPC demonstrates substantial performance gains at high speeds. The RMS and MA values of lateral error are reduced by approximately 80.0%, and the RMS and MA of the heading error decreased by 14.0% and 15.0%, respectively. These results confirm the effectiveness of the LSTM-based prediction model and NN-MPC strategy in improving trajectory tracking performance under demanding conditions.

**Figure 6:**
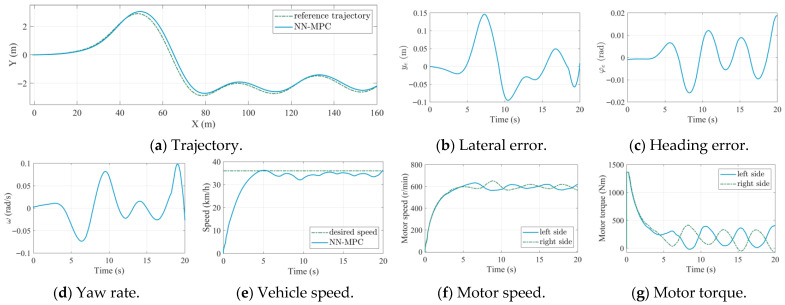
Simulation results of NN-MPC trajectory under a spline reference trajectory.

To further verify the robustness of the proposed NN-MPC, a random spline curve was used as a reference path, and the simulation results are shown in Figure 6. As shown in Figure 6a, although the reference path exhibits varying curvature, the actual path of the TUV closely followed the actual path, with a small lateral error and heading error, as depicted in Figure 6b,c. Additionally, the NN-MPC achieved high speed tracking accuracy, as shown in Figure 6e. The motor speed and torque are illustrated in Figure 6f,g. Due to the turning maneuvers, both the motor speed and torque vary continuously. The accurate trajectory tracking performance under the complex reference path highlights the robustness of NN-MPC.

### 4.2. Field Experiment

**Figure 7:**
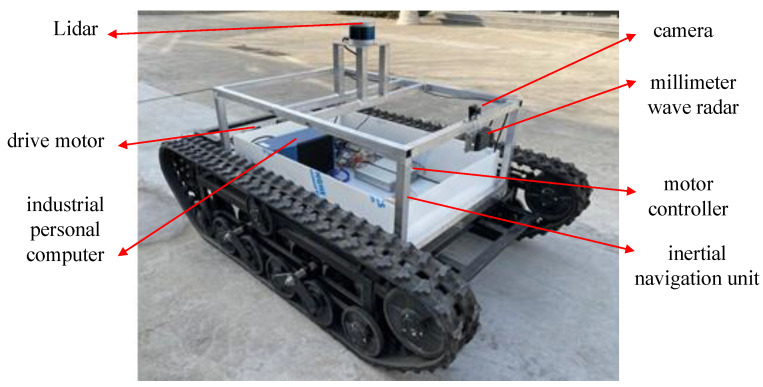
Dual-motor driven TUV in field experiment.

**Figure 8:**
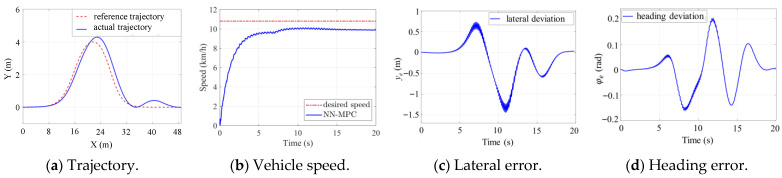

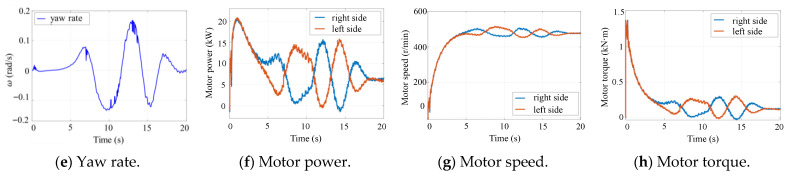
Field experiment results.

**Figure 9:**
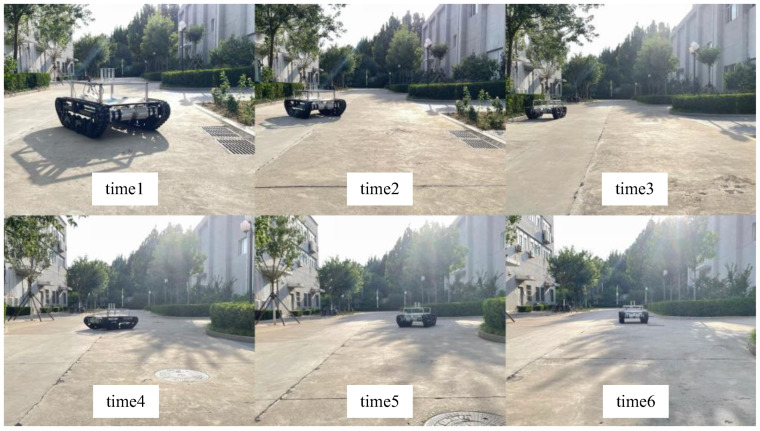
Field experiment scenario.

A field experiment was further conducted to verify the proposed NN-MPC using a scaled TUV, as shown in Figure 7. The parameters of the scaled TUV are listed in Table 8. The experiment results are shown in Figure 8, and the experiment scenario is shown in Figure 9.

Due to the limitations of the experimental site and to ensure safety during the field experiment, the trajectory tracking performance of the TUV was evaluated under a low-speed double-lane-change scenario. The desired speed of the TUV was set to 10.8 km/h, as shown in Figure 8b. The differences between the multi-dynamics model in RecurDyn and the real scaled TUV led to the reduced prediction accuracy of the trained LSTM-based vehicle model. Consequently, the lateral and yaw error are slightly larger than those observed in the simulations, as shown in Figure 8c,d. The maximum absolute values of lateral error and heading error were approximately 1.4 m and 0.16 rad, respectively. In addition, the actuator disturbance and sensor noise result in fluctuations in motor speed, torque, and power responses, as illustrated in Figure 8f–h. These factors also led to slight variations in the actual vehicle speed, which fluctuated around the desired value with a steady-state tracking error of approximate 0.2 m/s, as shown in Figure 8b. Despite these variations, the actual path of the TUV remained smooth, and the tracking accuracy was generally acceptable, as can be seen in Figure 8a.

Since the control horizon is relatively short (3 steps) and the prediction horizon is 15 steps, the optimization problem size remains compact. In addition, the LSTM-based prediction model is linearized around the current state and the control input at a previous time step, which further reduces computational overhead. The proposed NN-MPC algorithm was executed on an industrial computer equipped with a 16-core high-performance CPU. The control loop operated stably with a sampling period of 0.05 s, and no noticeable delay or computation bottlenecks were observed during the field experiment, confirming its real-time feasibility. In future work, we plan to deploy the controller on an embedded on-board chip to further evaluate its computational efficiency. Overall, these results confirm the practical feasibility and effectiveness of the proposed NN-MPC in real-world operating conditions.

## 5. Conclusions

In this article, a neural network-based MPC approach is proposed for trajectory tracking of a dual-motor-driven TUV. Considering the coupled lateral and longitudinal dynamics characteristics, a data-driven vehicle model based on a LSTM network was developed to predict the vehicle motion states in a subsequent time step through a sequence of history states and control inputs. Subsequently, an NN-MPC trajectory tracking controller was designed, employing the LSTM-based model as the prediction model to calculate the optimal torque. The various simulation results demonstrate that the NN-MPC achieves accurate trajectory tracking performance across different speeds and reference paths. The effectiveness of the proposed scheme is further validated through the field experiment.

For future work, the validation of the proposed control scheme under high-speed scenarios will be further investigated. In addition, coordinated control strategies that simultaneously consider longitudinal speed tracking, lateral path following, and handling stability will be explored to enhance overall motion performance. Furthermore, to address the differences between the simulation model and the real scaled tracked vehicle, the adaptation of the trained LSTM-based vehicle dynamics model to the real TUV will be studied.

## Figures and Tables

**Table 1:** Parameters of neural network.

Layer	Unit Number	Parameter Number
LSTM layer	32	5120
Fully Connected layer	16	528
Dropout layer	16	0
Output layer	5	85

**Table 2:** Dynamics simulation parameters.

Component Parts	Number	Moment of Inertia	Stiffness Coefficient	Damping Factor
Driving wheel	2	279,379 kg·mm^2^	18,000 N/mm	10 N·s/mm
Weight wheel	10	685,898 kg·mm^2^	12,000 N/mm	10 N·s/mm
Tractor wheel	6	5121 kg·mm^2^	15,000 N/mm	10 N·s/mm
Track plate	4	1989 kg·mm^2^	9000 N/mm	10 N·s/mm

**Table 3:** Comparative prediction results.

Model	Dynamic Model	DNN	LSTM
MSE	0.1002	0.0995	0.0429
MAE	0.2865	0.2853	0.1624

**Table 4:** Prediction results of parameter sensitivity analysis.

	Hidden Units: 16	Hidden Units: 32	Hidden Units: 64
state length: 10	0.0492	0.0489	0.0488
state length: 20	0.0435	0.0429	0.0429
state length: 30	0.0431	0.0428	0.0431

**Table 5:** Controller parameters.

Parameter	Value	Parameter	Value
The length of prediction horizon *N_P_*	15	The length of control horizon *N_P_*	3
The weight of lateral error *Q_ye_*	5	The weight of heading error *Q_φe_*	1
The weight of speed error *Q_ve_*	3	The weight of control input *R*	50

**Table 6:** Performance metrics of trajectory tracking under medium speeds.

	RMS*_l_*	RMS*_h_*	MA*_l_*	MA*_h_*
NN-MPC	0.2536 m	0.0550 rad	0.5644 m	0.1315 rad
Dyn-MPC	0.2884 m	0.0597 rad	0.6498 m	0.1428 rad

**Table 7:** Performance metrics of trajectory tracking under high speeds.

	RMS*_l_*	RMS*_h_*	MA*_l_*	MA*_h_*
NN-MPC	0.9297 m	0.1627 rad	2.4408 m	0.4211 rad
Dyn-MPC	4.6483 m	0.1914 rad	12.2041 m	0.4954 rad

**Table 8:** Parameters of TUV in field experiment.

Parameters	Value	Parameters	Value
Track center distance B	1 m	Wheel number 2n	10
Radius of active wheel $r_{z}$	0.15 m	Vehicle mass m	120 kg
Track grounding length L	1.7 m	Moment of inertia J	38.9 kg·m^2^

## Data Availability

The original contributions presented in this study are included in the article. Further inquiries can be directed to the corresponding author.

## Abbreviations

The following abbreviations are used in this manuscript:

LSTM	Long short-term memory
NN	Neural network
MPC	Model predictive control
TUV	tracked unmanned vehicles
